# Changes in metabolic syndrome status affect the incidence of end-stage renal disease in the general population: a nationwide cohort study

**DOI:** 10.1038/s41598-021-81396-0

**Published:** 2021-01-21

**Authors:** Eun Sil Koh, Kyung Do Han, Mee Kyoung Kim, Eun Sook Kim, Min-Kyung Lee, Ga Eun Nam, Oak-Kee Hong, Hyuk-Sang Kwon

**Affiliations:** 1 Division of Nephrology, Department of Internal Medicine, Yeouido St. Mary’s Hospital, College of Medicine, The Catholic University of Korea, Seoul, 07345 Republic of Korea; 2 Department of Statistics and Actuarial Science, Soongsil University, Seoul, 06978 Republic of Korea; 3 Division of Endocrinology and Metabolism, Department of Internal Medicine, Yeouido St. Mary’s Hospital, College of Medicine, The Catholic University of Korea, 10, 63-ro, Yeongdeungpo-gu, Seoul, 07345 Republic of Korea; 4 Division of Endocrinology and Metabolism, Department of Internal Medicine, Incheon St. Mary’s Hospital, College of Medicine, The Catholic University of Korea, Incheon, 21431 Republic of Korea; 5 Division of Endocrinology and Metabolism, Department of Internal Medicine, Myongji Hospital, Hanyang University Medical Center, Gyeonggi-do 10475, Goyang-Si, Republic of Korea; 6 Department of Family Medicine, Korea University Anam Hospital, Korea University College of Medicine, Seoul, 02841 Republic of Korea

**Keywords:** Endocrinology, Health care

## Abstract

Few studies have investigated the impact of a change in metabolic syndrome (MetS) components on clinical renal outcomes in the general population. Using nationally representative data from the Korean National Health Insurance System, 13,310,924 subjects who underwent two health examinations over 2 years and were free from end-stage renal disease (ESRD) from 2009 to 2012 were followed to the end of 2016. The subjects were divided into four groups according to the change in MetS components between the two visits over 2 years: no MetS (–/–), post-MetS (–/+), pre-MetS (+/–), and both MetS (+/+). After a median follow up of 5.11 years, 18,582 incident ESRD cases were identified. In the multivariate adjusted model, the hazard ratio (HR) and 95% confidence interval (CI) for the development of ESRD in the both-MetS (+/+) group compared with the no-MetS (–/–) group was 5.65 (95% CI, 5.42–5.89), which was independent of age, sex, and baseline estimated glomerular filtration rate. Additionally, the HR for the pre-MetS (+/–) group versus the no-MetS (–/–) group was 2.28 (2.15–2.42). In subgroup analysis according to renal function, the impact of a change in MetS on the incidence of ESRD was more pronounced in individuals with advanced renal dysfunction. Subjects with resolved MetS components had a decreased risk of ESRD, but not as low as those that never had MetS components. This provides evidence supporting the strategy of modulating MetS in the general population to prevent the development of ESRD.

## Introduction

Metabolic syndrome (MetS) is defined as the simultaneous occurrence of at least three of the following: abdominal obesity, elevated blood pressure (BP), elevated plasma glucose, and dyslipidaemia^1^. When these metabolic abnormalities are grouped together, they are associated with an increased risk of both diabetes and cardiovascular disease (CVD). Reports have demonstrated that MetS plays a critical role in the progression of chronic kidney disease (CKD) and CVD^2–4^. Since CKD is an established risk factor for CVD, it is unsurprising that MetS is a potential risk factor for CKD progression. Furthermore, diabetes and hypertension are the leading causes of the development of both CKD and end-stage renal disease (ESRD)^5^. Because impaired fasting glucose and elevated BP are included in the definition of MetS, investigations of the independent associations among the other three components of MetS and renal outcome are also important. In a recent cohort study performed in elderly Japanese women, both the presence of MetS and the number of MetS components were associated with a higher prevalence of CKD, and the MetS BP criterion may have enhanced the association^6^. In addition, Stefansson and collaborators^3^ confirmed MetS as an independent risk factor for an accelerated age-related glomerular filtration rate (GFR) decline in the general population. They showed that individuals with baseline MetS had a significantly faster mean decline of 0.30 mL/min/year compared with individuals without MetS in a multivariable adjusted linear regression model. However, other studies have reported a non-significant association between MetS and the risk of CKD development and cardiovascular risk^7–9^. Thus, controversies remain regarding this relationship.

MetS status can change over time through factors such as lifestyle modification or diverse comorbidities. Indeed, previous studies have observed considerable changes in the MetS status of individuals over follow-up periods^10–12^. Therefore, we investigated the associations between changes in MetS components and the incidence of ESRD in the general Korean population using a nationwide population-based cohort.

## Methods

### Data source and study population

**Figure 1:**
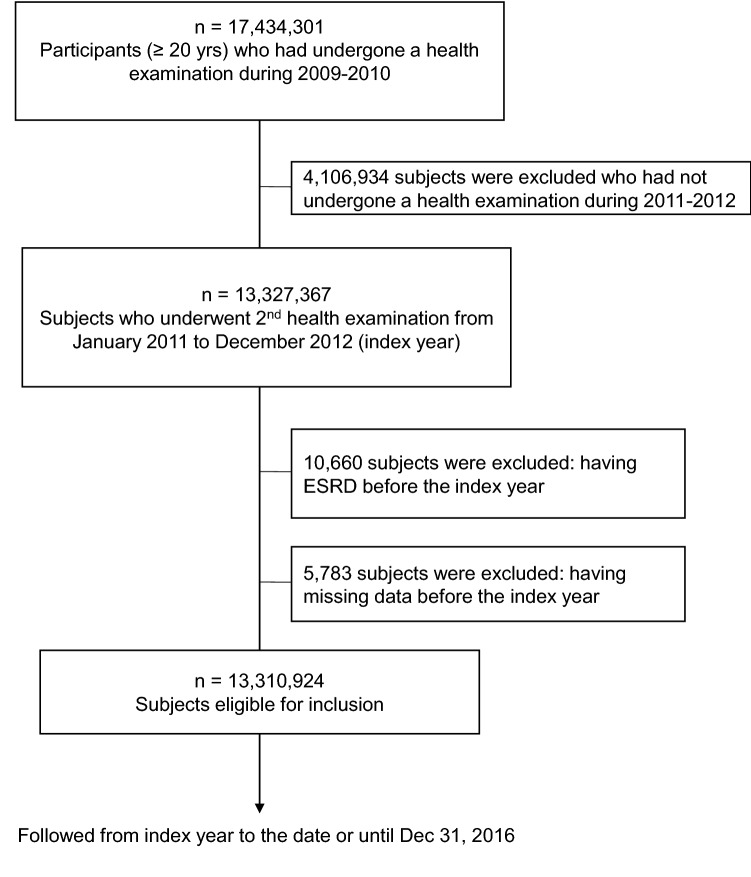
Study enrolment flow.

We used the National Health Insurance System (NHIS) database; the NIHS is government-managed and the only insurer providing regular health check-up programs to the public in South Korea. Those enrolled in the health insurance service are recommended to undergo health check-ups at least biennially. In the current study, we included 17,434,301 subjects aged ≥ 20 years who underwent a health examination between 2009 and 2010. Of these, we included 13,327,367 subjects with two visits over 2 years; underwent a second health examination during 2011–2012 (index year). We excluded 10,660 subjects having ESRD and 5,783 subjects having missing data prior to the index year. Finally, the study included 13,310,924 subjects (Fig. 1). All research was performed in accordance with relevant guidelines and regulations.

### Data collection

Comorbidities were defined mainly by a combination of past history (ICD code and self-reported) and use of medication history for the corresponding disease. The presence of hypertension was defined according to the presence of at least one claim per year under ICD-10 codes I10 or I11 and at least one claim per year for the prescription of an antihypertensive agent or systolic/diastolic BP ≥ 140/90 mmHg^13,14^. The presence of diabetes mellitus was defined according to the following criteria: (1) at least one claim per year under ICD–10 codes E10–14 and at least one claim per year for the prescription of antidiabetic medication, or (2) fasting glucose level ≥ 126 mg/dL^13^. Blood samples for the measurement of serum glucose, creatinine (Cr), total cholesterol, triglyceride (TG), high-density lipoprotein cholesterol (HDL-C), and low-density lipoprotein cholesterol (LDL-C) levels were drawn after fasting overnight. Estimated GFR was calculated using the four variable modification of diet in renal disease (MDRD) formula: eGFR = 175 × serum Cr (mg/dL) ^–1.154^ × age (y) ^– 0.203^ × (0.742 if female)^15,16^.

### Diagnosis of metabolic syndrome and definition of the change in metabolic syndrome components

Based on the National Cholesterol Education Program’s Adult Treatment Panel III^13^, we defined MetS as at least three of the five following criteria: (1) abdominal obesity, as defined by a waist circumference (WC) ≥ 90 cm for men and ≥ 85 cm for women; (2) elevated TG levels (≥ 150 mg/dL); (3) decreased HDL-C levels (< 40 mg/dL for men and < 50 mg/ dL for women); (4) elevated BP (systolic BP ≥ 130 mmHg, diastolic BP ≥ 85 mmHg) or treatment for previously diagnosed hypertension; and (5) elevated fasting plasma glucose levels (≥ 100 mg/dL) or previously diagnosed diabetes mellitus type 2.

We divided the individuals into four groups according to the changes in MetS components over the 2-year interval between the first and second health exam: no MetS (–/–), post-MetS (–/+), pre-MetS (+/–), and both MetS (+/+). Individuals in the no-MetS (–/–) group had no diagnosis of MetS at either exam, those in the post-MetS (–/+) group were newly diagnosed with MetS at the second visit, those in the pre-MetS (+/–) group resolved MetS after the first exam, and the both-MetS (+/+) group had MetS that persisted at both exams.

### Study outcome and follow-up

The study outcome was newly diagnosed ESRD. We defined incident ESRD using the combination of ICD-10 code (N18-19, Z49, Z94.0, Z99.2) and initiation of renal replacement therapy and/or kidney transplantation (KT) during hospitalisation^13^. The Korean Health Insurance Review and Assessment Service reimburses all medical care expenses for dialysis. Patients with ESRD are also registered as special medical aid beneficiaries^17^. Therefore, we could include each ESRD patient in the whole South Korean population and analyse the data for all ESRD patients who started dialysis^17,18^. The codes for treatment or medical expense claims were O7011-O7020 or V001 for haemodialysis, O7071-O7075 or V003 for peritoneal dialysis, and R3280 for KT^14^. We excluded individuals without previous CKD who had a transplant or dialysis code on the same date as an acute renal failure code. Subjects on continuous renal replacement therapy or acute peritoneal dialysis were also excluded^17,18^. The study population was followed from baseline at the index year to the date of ESRD diagnosis or December 31, 2016, whichever came first.

### Statistical analysis

Baseline characteristics are presented as means ± standard deviation or n (%). The incidence of primary outcomes was calculated by dividing the number of incident cases by the total follow-up duration (person-years)^19^. The disease-free probability of primary outcomes according to the change in MetS components was calculated using Kaplan–Meier curves, and a log-rank test was performed to analyse differences among the groups. Hazard ratios (HRs) and 95% confidence intervals (CIs) for ESRD were calculated using a Cox proportional hazards model for each category. Model 1 was adjusted for number of MetS components at 1st visit, age and sex. Model 2 was additionally adjusted for smoking status, alcohol consumption and exercise with variables from model 1. In model 3, we further adjusted for eGFR in addition to variables from model 2. Statistical analyses were performed using SAS ver. 9.4 (SAS Institute Inc., Cary, NC, USA), and a *P* value < 0.05 was considered to indicate significance.

### Ethics approval and consent to participate

This study was conducted according to the Declaration of Helsinki. Informed consent requirement was waived by the Ethics Committee of The Catholic University of Korea, because personal identifying information was not accessed. The study was approved by the Institutional Review Board of The Catholic University of Korea (No. SC18ZESI0053).

## Results

### Characteristics of each group according to the change in MetS components

Table 1 shows the demographics of the study population according the change in MetS components. The proportion of subjects in the no-MetS (–/–) group was 61.3%, compared with 10.8%, 8.3%, and 19.5% in the post-MetS (–/+), pre-MetS (+/–), and both-MetS (+/+) groups, respectively. Individuals in the both-MetS (+/+) group were generally older, female, had a higher body mass index (BMI) and a larger WC, exercised less, had a higher prevalence of diabetes and hypertension, increased TG levels, and lower HDL-C and LDL-C levels (Table 1).

**Table 1:** Demographics of the study population according the change in MetS components.

	No-MetS (–/–)	Post-MetS (–/+)	Pre-MetS (+/–)	Both-MetS (+/+)
	(n = 8,167,333)	(n = 1,438,425)	(n = 1,105,622)	(n = 2,599,544)
**At second visit (index year)**				
Age, years	46.4 ± 13.1	53.7 ± 12.9	54.3 ± 12.9	59.0 ± 12.0
Male, n (%)	4,297,945 (52.6)	820,951 (57.1)	643,050 (58.2)	1,288,805 (49.6)
BMI, kg/ m^2^	22.8 ± 2.8	25.1 ± 3.0	24.7 ± 2.9	26.0 ± 3.1
Body weight, kg	61.6 ± 10.6	67.3 ± 12.1	66.2 ± 11.7	68.2 ± 12.5
Height, cm	164.2 ± 8.9	163.2 ± 9.6	163.2 ± 9.6	161.4 ± 9.7
WC, cm	77.4 ± 8.0	85.0 ± 7.8	82.8 ± 7.7	87.3 ± 8.1
Current smoker, n (%)	1,856,325 (22.8)	342,407 (23.8)	263,912 (23.9)	494,569 (19.0)
Heavy drinker, n (%)	429,794 (5.3)	106,839 (7.5)	78,270 (7.1)	170,747 (6.6)
Regular exercise, n (%)	4,579,387 (56.1)	750,991 (52.2)	591,503 (53.5)	1,273,424 (49.0)
Income lower 25%, n (%)	1,754,695 (21.5)	325,070 (22.6)	252,501 (22.8)	603,356 (23.2)
Diabetes, n (%)	113,671 (1.4)	106,124 (7.4)	74,375 (6.7)	681,161 (26.2)
Hypertension, n (%)	601,039 (7.4)	422,136 (29.4)	264,618 (23.9)	1,488,282 (57.3)
eGFR, mL/min/1.73 m^2^	91.6 ± 36.6	87.8 ± 35.0	88.2 ± 36.4	84.7 ± 34.4
Fasting glucose, mg/dL	92.1 ± 14.3	104.3 ± 22.8	98.56 ± 22.33	112.69 ± 31.97
Systolic BP, mmHg	118.3 ± 13.4	129.4 ± 13.8	124.75 ± 13.9	130.8 ± 14.6
Diastolic BP, mmHg	74.0 ± 9.3	80.3 ± 9.6	77.62 ± 9.39	80.16 ± 9.92
TC, mg/dL	192.0 ± 33.2	205.1 ± 40.0	201.93 ± 36.02	197.37 ± 42.12
TG, mg/dL	93.7 (93.7–93.8)	158.0 (157.9–158.1)	122.91 (122.8–123.02)	163.19 (163.09–163.3)
HDL-C, mg/dL	58.0 ± 14.1	50.2 ± 14.1	53.1 ± 13.8	49.1 ± 13.9
LDL-C, mg/dL	113.2 ± 35.2	120.4 ± 41.9	122.0 ± 37.6	112.5 ± 44.0
**At first visit**				
BMI, kg/ m^2^	22.7 ± 2.7	24.73 ± 2.83	25.05 ± 2.88	26.08 ± 3.07
WC, cm	77.0 ± 8.0	82.74 ± 7.55	84.97 ± 7.64	87.25 ± 7.94
eGFR, mL/min/1.73 m^2^	89.6 ± 44.2	86.39 ± 38.56	85.56 ± 37.99	83.04 ± 36.12
Fasting glucose, mg/dL	91.6 ± 14.5	97.3 ± 21.66	104.33 ± 23.23	111.91 ± 32.12
Systolic BP, mmHg	118.1 ± 13.3	124.68 ± 14.11	129.76 ± 13.75	131.38 ± 14.78
Diastolic BP, mmHg	73.9 ± 9.2	77.79 ± 9.48	80.50 ± 9.57	80.86 ± 9.98
TC, mg/dL	190.0 ± 33.0	205.33 ± 36.46	203.88 ± 38.4	203.91 ± 42.2
TG, mg/dL	92.8 (92.8–92.8)	125.71 (125.61–125.8)	162.6 (162.4–162.8)	170.9 (170.8–171.0)

Data are expressed as the mean ± SD, median value (interquartile range) or n (%).

*MetS* metabolic syndrome, *BMI* body mass index, *WC* waist circumference, *BP* blood pressure, *TC* total cholesterol, *TG* triglyceride, *HDL-C* high-density lipoprotein cholesterol, *LDL-C* low-density lipoprotein cholesterol, *eGFR* estimated glomerular filtration rate.

### Association between the presence of each MetS component and the risk of ESRD

After a median follow up of 5.11 years, 18,582 incident ESRD cases were identified. We analysed the impact of the presence of each MetS component on the incidence of ESRD (Table 2). Subjects with MetS had a hazard ratio (HR) of 3.51 (95% confidence intervals (CI), 3.40–3.63) for incident ESRD compared with those without MetS after adjusting for age and sex (model 1). After additionally adjusted for smoking status, alcohol consumption and exercise with variables from model 1 (model 2), subjects with MetS had a HR of 4.07 (95% CI, 3.93–4.22); in model 3 with further adjusting for eGFR, those had a HR of 3.57 (95% CI, 3.45–3.70). All MetS criteria increased the risk of ESRD development, among which the BP criterion had the highest HR. That is, subjects with the BP criterion had a 4.57-fold increased risk of ESRD development compared with those without the BP criterion. Among the five MetS, WC had the lowest HR of 1.55 (95% CI, 1.49–1.61) versus those without central obesity after adjusting for all confounding variables (model 3).

**Table 2:** Adjusted hazard ratios, 95% confidence intervals, and incidence rates of end-stage renal disease development according to the presence of each MetS component.

	N	Events (n)	Follow-up duration (person-years)	Incidence rate (per 1000 person-years)	Adjusted hazard ratios ( 95% confidence intervals)		
					Model 1	Model 2	Model 3
**WC criterion**							
No	10,544,298	12,275	52,652,426	0.23	1 (ref.)	1 (ref.)	1 (ref.)
Yes	2,766,626	6,307	13,733,667	0.46	1.45 (1.41, 1.49)	1.51 (1.45, 1.57)	1.55 (1.49, 1.61)
**BP criterion**							
No	7,181,413	2,252	35,937,081	0.06	1 (ref.)	1 (ref.)	1 (ref.)
Yes	6,129,511	16,330	30,449,013	0.54	4.85 (4.63, 5.07)	5.23 (5.00, 5.48)	4.57 (4.36, 4.78)
**Glucose criterion**							
No	8,904,300	6,473	44,579,195	0.15	1 (ref.)	1 (ref.)	1 (ref.)
Yes	4,406,624	12,109	21,806,898	0.56	2.53 (2.46, 2.61)	2.59 (2.51, 2.67)	2.26(2.19, 2.33)
**TG criterion**							
No	8,303,071	6,610	41,430,791	0.16	1 (ref.)	1 (ref.)	1 (ref.)
Yes	5,007,853	11,972	24,955,302	0.48	2.25 (2.19, 2.32)	2.29 (2.22, 2.36)	2.1 (2.03,2.16)
**HDL-C criterion**							
No	9,204,479	6662	46,048,303	0.15	1 (ref.)	1 (ref.)	1 (ref.)
Yes	4,106,445	11,920	20,337,791	0.59	3.11 (3.01, 3.20)	2.99 (2.90, 3.09)	2.66 (2.58, 2.75)
**MetS**							
No	9,272,955	5498	46,399,629	0.12	1 (ref.)	1 (ref.)	1 (ref.)
Yes	4,037,969	13,084	19,986,465	0.65	3.51 (3.40, 3.63)	4.07 (3.93, 4.22)	3.57 (3.45, 3.70)

Model 1, adjusted for No. of MetS components at 1st visit, age and sex; Model 2, Model 1 plus adjusted for smoking, drinking and exercise; Model 3, Model 2 plus adjusted for estimated GFR.

*WC* waist circumference,* BP* blood pressure,* TG* triglyceride,* HDL-C* high-density lipoprotein cholesterol; MetS, metabolic syndrome.

Consistent with previous reports, the risk of ESRD development increased progressively as the number of components of MetS increased from one to five criteria; subjects with five criteria of MetS had an ~ 24-fold increased risk of developing ESRD compared with subjects with no criteria. These results showed a similar pattern as the data from the first visit (Supplementary Table S1).

### Risk of ESRD according to the change in each MetS component

We assessed the associations between the change in each MetS component and the risk of ESRD development (Table 3). The HR and 95% CI comparing the both-MetS (+/+) group with the no-MetS (–/–) group was 5.09 (95% CI, 4.90–5.28) for ESRD development in model 1, and 6.69 (95% CI, 6.42–6.97) in model 2 and 5.65 (95% CI, 5.42–5.89) in model 3, respectively. In terms of WC, the HR for ESRD in the both-WC (+/+) group was 1.95 (95% CI, 1.86–2.05), compared with 1.53 (1.45–1.61) in the pre-WC (+/–) group after adjusting for all confounding variables including baseline eGFR (model 3). The HR of individuals in the both-BP (+/+) group was 7.02 (95% CI, 6.62–7.44), whereas that of individuals in the resolved-BP (+/–) group was 1.94 (1.78–2.11) in model 3. Also, the results with other MetS criteria—including glucose, TG and HDL-C showed the similar pattern (Table 3). However, the change in obesity, which was defined as a BMI > 25 kg/m^2^, had a particularly interesting effect. Unlike other MetS criteria, subjects in the resolved-obesity (+/–) group had a higher risk of ESRD development than persistently obese subjects when the no-obesity (–/–) group was used as the reference.

**Table 3:** Adjusted hazard ratios, 95% confidence intervals, and incidence rates of end-stage renal disease development according to the change of each MetS component.

		N	Events (n)	Follow-up duration (person-years)	Incidence rate (per 1000 person-years)	Adjusted hazard ratios ( 95% confidence intervals)		
						Model 1	Model 2	Model 3
**WC criterion**	No (–/–)	9,652,099	10,380	48,230,672	0.22	1 (ref.)	1 (ref.)	1 (ref.)
	Post (–/+)	1,010,018	1705	5,015,280	0.34	1.23 (1.17,1.30)	1.36 (1.29, 1.44)	1.44 (1.36,1.52)
	Pre (+/–)	892,199	1895	4,421,754	0.43	1.38 (1.32,1.45)	1.49 (1.42, 1.57)	1.53 (1.45,1.61)
	Both (+/+)	1,756,608	4602	8,718,387	0.53	1.66 (1.60,1.72)	1.93 (1.84, 2.02)	1.95 (1.86, 2.05)
**BP criterion**	No (–/–)	5,746,514	1374	28,742,096	0.05	1 (ref.)	1 (ref.)	1 (ref.)
	Post (–/+)	1,685,406	1314	8,424,416	0.16	2.32 (2.15, 2.50)	2.50 (2.32, 2.70)	2.4 (2.22, 2.59)
	Pre (+/–)	1,434,899	878	7,194,985	0.12	1.84 (1.69, 2.00)	1.95 (1.79, 2.13)	1.94 (1.78, 2.11)
	Both (+/+)	4,444,105	15,016	22,024,597	0.68	7.21 (6.81, 7.63)	8.25 (7.78, 8.74)	7.02 (6.62, 7.44)
**Glucose criterion**	No (–/–)	7,347,391	4918	36,791,423	0.13	1 (ref.)	1 (ref.)	1 (ref.)
	Post (–/+)	1,760,736	1742	8,761,835	0.20	1.13 (1.07,1.20)	1.17 (1.10, 1.23)	1.06 (1.01, 1.12)
	Pre (+/–)	1,556,909	1555	7,787,772	0.20	1.16 (1.094,1.23)	1.191 (1.12, 1.26)	1.15 (1.09, 1.22)
	Both (+/+)	2,645,888	10,367	13,045,063	0.79	3.42 (3.30, 3.54)	3.55 (3.43, 3.68)	3.02 (2.91, 3.13)
**TG criterion**	No (–/–)	6,899,908	4699	34,438,940	0.14	1 (ref.)	1 (ref.)	1 (ref.)
	Post (–/+)	1,678,415	2332	8,385,458	0.28	1.61 (1.53, 1.69)	1.64 (1.56, 1.73)	1.59 (1.51, 1.67)
	Pre (+/–)	1,403,163	1911	6,991,851	0.27	1.49 (1.41, 1.57)	1.52 (1.44, 1.61)	1.50 (1.42,1.58)
	Both (+/+)	3,329,438	9640	16,569,844	0.58	2.88 (2.78, 2.98)	2.99(2.89, 3.10)	2.67 (2.57, 2.77)
**HDL-C criterion**	No (–/–)	7,854,976	4845	39,324,738	0.12	1 (ref.)	1 (ref.)	1 (ref.)
	Post (–/+)	1,620,717	3076	8,041,271	0.38	2.53 (2.42, 2.65)	2.45 (2.34, 2.57)	2.36 (2.25,2.47)
	Pre (+/–)	1,349,503	1817	6,723,565	0.27	1.92 (1.82, 2.02)	1.86 (1.77,1.97)	1.84 (1.74, 1.94)
	Both (+/+)	2,485,728	8844	12,296,520	0.72	4.31 (4.15, 4.47)	4.17 (4.02, 4.33)	3.55 (3.41, 3.68)
**Met S**	No (–/–)	8,167,333	3930	40,896,957	0.10	1 (ref.)	1(ref.)	1 (ref.)
	Post (–/+)	1,438,425	2210	7,147,323	0.31	2.23 (2.12, 2.36)	2.68 (2.54, 2.82)	2.54 (2.40, 2.68)
	Pre (+/–)	1,105,622	1568	5,502,672	0.28	1.98 (1.87, 2.10)	2.32 (2.19, 2.46)	2.28 (2.15, 2.42)
	Both (+/+)	2,599,544	10,874	12,839,141	0.85	5.09 (4.90, 5.28)	6.69 (6.42, 6.97)	5.65 (5.42, 5.89)
**Obesity**	No (–/–)	8,251,129	10,223	41,158,447	0.25	1 (ref.)	1 (ref.)	1 (ref.)
	Post (–/+)	753,917	1065	3,760,971	0.28	1.13 (1.06, 1.20)	1.06 (0.99, 1.14)	1.06 (0.99, 1.14)
	Pre (+/–)	650,083	1391	3,223,253	0.43	1.38 (1.31, 1.46)	1.34 (1.26, 1.42)	1.32 (1.25, 1.40)
	Both (+/+)	3,655,795	5903	18,243,423	0.32	1.20 (1.16, 1.23)	1.10 (1.04, 1.16)	1.14 (1.08, 1.20)

Model 1, adjusted for No. of MetS components at 1st visit, age and sex; Model 2, Model 1 plus adjusted for smoking, drinking and exercise; Model 3, Model 2 plus adjusted for estimated GFR.

*WC* waist circumference,* BP* blood pressure,* TG* triglyceride,* HDL-C* high-density lipoprotein cholesterol,* MetS* metabolic syndrome.

### Risk of ESRD according to the change in number of MetS components

**Figure 2:**
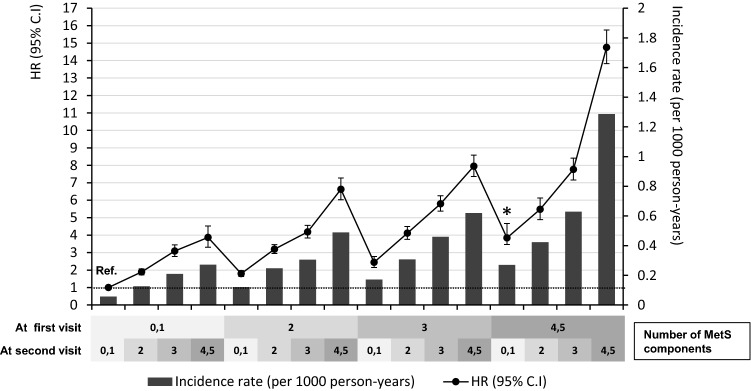
Adjusted hazard ratios, 95% confidence intervals, and incidence rates of end-stage renal disease according to changes in the number of metabolic syndrome components as reference with the subjects with 0–1 MetS components at both exams over two years. The model was adjusted for metabolic syndrome components at first visit, age, sex, smoking, alcohol consumption, exercise, and estimated glomerular filtration rate. *The subjects whose the number of MetS components decreased from 4–5 even to 0–1, the HR for the risk of ESRD remained 3.84 (95% CI, 3.17–4.67) compared with subjects who persistently had 0–1 MetS components over two years.

Figure 2 shows the risk of incident ESRD according to the change in number of MetS components when compared with subjects with 0–1 MetS components at both exams over two years. As the number of MetS components increase at second visit (index year), the risk of incident ESRD increase regardless of the number of MetS components at first visit. As the number of MetS components decreased from 4–5 to 3, 2, and 0–1 over two years, the HR for incident ESRD decreased sequentially from 14.76 (95% CI, 13.83–15.75) to 7.76 (7.16–8.41), 5.48 (4.90–6.13), and 3.84 (3.17–4.67), respectively. However, it did not show absolute recovery to the same degree of risk observed in individuals with 0–1 MetS components at both visits. Thus, the subject whose the number of MetS components decreased from 4–5 even to 0–1 (expressed with asterisk in Fig. 2), the HR for the risk of incident ESRD remained 3.84 (95% CI, 3.17–4.67) compared with subjects who persistently had 0–1 MetS components over two years (reference).

### Stratified analysis for risk of ESRD in different renal function subgroups

We conducted subgroup analyses in each group with different renal functions. When we divided subjects into three subgroups (eGFR < 60 mL/min/1.73 m^2^, 60 mL/min/1.73 m^2^ < eGFR < 90 mL/min/1.73 m^2^, and > 90 mL/min/1.73 m^2^) the risk of ESRD was higher in groups with more advanced renal dysfunction (Supplementary Table S2). That is, individuals meeting all five MetS criteria exhibited an ~ 85-fold increased risk of ESRD in subgroups with an eGFR < 60 mL/min 1.73 m^2^ compared to those without any MetS criteria. By contrast, in the eGFR 60–90 mL/min/1.73 m^2^ and eGFR > 90 mL/min 1.73 m^2^ subgroups, subjects with all five MetS criteria had HRs of 9.30 (95% CI, 7.84–11.03) and 5.01 (3.95–6.36), respectively.

In terms of changes in MetS components, the impact of MetS over two years was stronger in the more advanced renal dysfunction subgroups (Table 4). The HR of individuals in the both-MetS (+/+) group was 2.49 (95% CI, 2.20–2.82) versus the no-MetS (–/–) group in the subgroup of patients with an eGFR > 90 mL/min/1.73 m^2^. The HRs of subjects in the both-MetS (+/+) group versus the no-MetS (–/–) group were 3.12 (2.88–3.39) and 7.95 (7.50–8.42) in the subgroups with an eGFR of 60–90 mL/min/1.73 m^2^ and < 60 mL/min/1.73 m^2^, respectively. In addition, the difference in the risk of ESRD between the both-MetS (+/+) and pre-MetS (+/–) groups was larger in the advanced renal dysfunction subgroups.

**Table 4:** Adjusted hazard ratios, 95% confidence intervals, and incidence rates of end-stage renal disease development according to the change of MetS component in different renal function groups.

eGFR (ml/min 1.73 m^2^)	Met S	N	Events (n)	Follow-up duration (person-years)	Incidence rate (per 1000 person-years)	Adjusted hazard ratios ( 95% confidence intervals)		
						Model 1	Model 2	Model 3
** ≥ 90**								
	No (–/–)	3,860,370	743	19,327,628	0.04	1 (ref.)	1 (ref.)	1 (ref.)
	Post (–/+)	569,066	238	2,827,416	0.08	1.53 (1.32, 1.77)	1.70 (1.46, 1.98)	1.70 (1.46, 1.98)
	Pre (+/–)	443,490	165	2,207,881	0.07	1.29 (1.09, 1.53)	1.42 (1.19, 1.68)	1.42 (1.19,1.68)
	Both (+/+)	886,095	604	4,386,917	0.14	2.10 (1.88, 2.35)	2.49 (2.20, 2.82)	2.49 (2.20, 2.82)
**60–90**								
	No (–/–)	4,033,388	1287	20,186,181	0.06	1 (ref.)	1 (ref.)	1 (ref.)
	Post (–/+)	782,638	578	3,892,718	0.15	1.70 (1.54, 1.88)	1.92 (1.73, 2.12)	1.88 (1.70, 2.08)
	Pre (+/–)	598,265	392	2,981,306	0.13	1.45 (1.29,1.62)	1.61 (1.43, 1.80)	1.59 (1.41, 1.78)
	Both (+/+)	1,438,726	1935	7,125,683	0.27	2.66 (2.47, 2.86)	3.21 (2.96, 3.48)	3.12 (2.88, 3.39)
** < 60**								
	No (–/–)	273,575	1900	1,383,148	1.37	1 (ref.)	1 (ref.)	1 (ref.)
	Post (–/+)	86,721	1394	427,189	3.26	2.30 (2.14, 2.46)	2.85 (2.65, 3.06)	3.32 (3.09,3.57)
	Pre (+/–)	63,867	1011	3,134,865	3.23	2.29 (2.12, 2.47)	2.75 (2.55, 2.98)	3.12(2.89, 3.38)
	Both (+/+)	274,723	8335	1,326,542	6.28	4.70 (4.45, 4.95)	6.47 (6.11, 6.85)	7.95 (7.50, 8.42)

Model 1, adjusted for No. of MetS components at 1st visit, age and sex; Model 2, Model 1 plus adjusted for smoking, drinking and exercise; Model 3, Model 2 plus adjusted for estimated GFR.

## Discussion

The present study demonstrated the impact of a change in MetS status over 2 years on the incidence of ESRD as well as per se MetS in the nationwide 13.3 million study population, which is about 26% of total 50 million South Koreans. A total of 19.1% of individuals experienced changes in MetS status (i.e. the post-MetS (+/–) and pre-MetS (–/+) groups). Compared with the no-MetS (–/–) group, subjects in the post-MetS (–/+) group had an ~ 2.5-fold increased risk of ESRD at the second visit after a median follow up of 5.11 years from the index year. Of particular interest, the present study included a comparison of the risk between subjects in the pre-MetS (+/–) and both-MetS (+/+) groups. Although the pre-MetS group had a reduced risk of ESRD compared with subjects with the both-MetS (+/+) group, the risk did not decline to the degree observed in subjects with no MetS (–/–). Furthermore, the HR for the development of ESRD remained 2.28 (95% CI, 2.15–2.42). Even when the number of MetS components declined from 2 to 0–1, the HR for ESRD remained 1.80 (1.63–1.97) compared with individuals without MetS components. Thus, an initial metabolic change over 2 years had a distinct impact on renal outcomes for a median follow-up period of 5.11 years. This legacy effect could be associated with the concept of “metabolic memory”, where transient intensive metabolic controls create a “memory” for the improvement of microvascular outcomes^20–22^. Metabolic controls include the regulation of glucose, BP, and dyslipidaemia, as well as reduced WC^21^. The findings imply that early successful management of MetS can attenuate renal damage, and suggest that intensive interventions should be initiated as early as possible to prevent ESRD^23^.

Data published in the last decade have revealed that MetS also affects the risk of CKD progression^4,24–28^. The current study confirmed that the risk of ESRD increased progressively as the number of MetS components increased from 1 to 5, with HRs that were increased by 2.8-, 4.8-, 8.2-, 14.5-, and 24.8-fold compared with individuals with no-MetS components. Thus, multiple metabolic components had synergistic rather than additive effects of the risk of ESRD. The exact mechanism explaining the link between MetS and kidney disease has not been completely elucidated, although proposed pathophysiological factors include hyperfiltration, insulin resistance, adipokines, endothelial dysfunction, renin–angiotensin–aldosterone-system activation, and oxidative stress^1,11^.

Another interesting observation was that a change in obesity, defined as a BMI > 25 kg/ m^2^, exhibited a different pattern from the changes in other MetS criteria. Compared with the no-obesity (–/–) group, subjects in the pre-obesity (+/–) group had a higher risk of developing ESRD than those in the both-obesity (+/+) group. These trends were inconsistent with the change in the WC criterion; the HRs for developing ESRD in the both-WC (+/+) and pre-WC (+/–) groups were 1.95 and 1.53, respectively. Therefore, subjects who reduced their WC over 2 years had a lower risk of ESRD compared with those with sustained central obesity. However, subjects who reduced their BMI to < 25 kg/ m^2^ over 2 years had a higher risk of ESRD compared with those with a sustained BMI ≥ 25 kg/ m^2^. These results were consistent with previous studies demonstrating that a lower BMI resulted in poor outcomes in patients with CKD or ESRD^26–29^. BMI correlates well with adiposity, but it does not reflect the fat distribution pattern and can be affected by muscle mass^30^. Therefore, the opposing impact of a change in BMI and WC on ESRD could be explained by the poor prognosis of patients with both extreme malnutrition and excessive adiposity, with U-curve or J-curve patterns in CKD patients^31–36^.

A previous study demonstrated that early intervention in MetS could slow CKD progression in patients with early-stage CKD^11^, and established the risk of MetS change on renal function in individuals with early-stage CKD. However, in the subgroup analysis according to renal function in the present study, the impact of a change in MetS components on the incidence of ESRD was stronger in more advanced CKD groups. In the subgroup of patients with an eGFR < 60 mL/min/1.73 m^2^, individuals in the both-MetS (+/+) and pre-MetS (+/–) groups had HRs for ESRD development of 7.95 and 3.12, respectively, compared with the no-MetS (–/–) group. These findings support the hypothesis that the impact of MetS on ESRD differs according to the degree of renal function and that interventions to achieve active control of MetS may result in better renal outcomes, even in subjects with advanced CKD^28,37^.

Several findings from the present study warrant further consideration. First, the study followed an observational design; therefore, inferences of causality should not be made regarding individual changes in MetS components and declining renal function. Second, the assessment of renal function was limited, and no information on proteinuria was available. Calculating estimated GFR using a serum Cr-based MDRD formula is less accurate for subjects with a higher GFR than for those with a lower GFR. Also, the formula may not be suitable for use outside of American white and black populations. Despite these limitations, this is the first nationally representative, population-based study to evaluate changes in MetS components as risk factors for ESRD, and the results verify the possibility of a potential protective effect of combining individual MetS improvements using either medications or lifestyle modifications to slow the progression of CKD. A future intervention study could help isolate the impact of changes in MetS components as a risk factor for renal outcome. It is noteworthy to emphasise the need to regulate these metabolic risk factors with active interventions, particularly lifestyle modifications, to prevent the development of ESRD.

## Supplementary information

Supplementary Information.

Supplementary Information.
