# Supplementary material for: Changes in metabolic syndrome status affect the incidence of end-stage renal disease in the general population: a nationwide cohort study
Source: Sci Rep. 2021 Jan 21;11:1957. doi: 10.1038/s41598-021-81396-0 (PMC7820283; doi:10.1038/s41598-021-81396-0)
Supplement: Supplementary file 1 — Supplementary Information. [file 41598_2021_81396_MOESM1_ESM.docx]

**Changes in metabolic syndrome status affect the incidence of end-stage renal disease in the general population: a nationwide cohort study**

Eun Sil Koh^1^, Kyung Do Han^2^, Mee Kyoung Kim^3^, Eun Sook Kim^4^, Min-Kyung Lee^5^, Ga Eun Nam^6^, Oak-Kee Hong^3^ and Hyuk-Sang Kwon^3,*^

^1^Division of Nephrology, Department of Internal Medicine, Yeouido St. Mary’s Hospital, College of Medicine, The Catholic University of Korea, Seoul 07345, Republic of Korea

^2^Department of Statistics and Actuarial Science, Soongsil University, Seoul 06978, Republic of Korea

^3^Division of Endocrinology and Metabolism, Department of Internal Medicine, Yeouido St. Mary’s Hospital, College of Medicine, The Catholic University of Korea, Seoul 07345, Republic of Korea

^4^Division of Endocrinology and Metabolism, Department of Internal Medicine, Incheon St. Mary’s Hospital, College of Medicine, The Catholic University of Korea, Incheon 21431, Republic of Korea

^5^Division of Endocrinology and Metabolism, Department of Internal Medicine, Myongji Hospital, Hanyang University Medical Center, Goyang-Si, Gyeonggi-do 10475, Republic of Korea

^6^Department of Family Medicine, Korea University Anam Hospital, Korea University College of Medicine, Seoul 02841, Republic of Korea

**Word Count:** 3,186 words

**Key words:** Metabolic syndrome; ESRD; Change

***Corresponding author:** Hyuk-Sang Kwon, MD, PhD

Address: Division of Endocrinology and Metabolism, Department of Internal Medicine, Yeouido St. Mary’s Hospital, College of Medicine, The Catholic University of Korea, 10, 63-ro, Yeongdeungpo-gu, Seoul 07345, Republic of Korea

Tel: +82-2-3779-1039, Fax: +82-2-780-3132, E-mail: [drkwon@catholic.ac.kr](mailto:drkwon@catholic.ac.kr)

| **Supplementary Table S1 Adjusted hazard ratios, 95% confidence intervals, and incidence rates of end-stage renal disease development according to number of MetS component** | | | | | | | |
| --- | --- | --- | --- | --- | --- | --- | --- |
| **No. of MetS components** | **N** | **Events (n)** | **Follow-up duration (person-years)** | **Incidence rate (per 1000 person-years)** | Adjusted hazard ratios ( 95% confidence intervals) | | |
|  |  |  |  |  | **Model 1** | **Model 2** | **Model 3** |
| **At first visit** |  |  |  |  |  |  |  |
| **0** | 3,359,234 | 629 | 16,829,262 | 0.04 | 1 (ref.) | 1 (ref.) | 1 (ref.) |
| **1** | 3,448,114 | 2,181 | 17,258,666 | 0.13 | 2.32 (2.12, 2.53) | 2.63 (2.41, 2.88) | 2.63 (2.41, 2.88) |
| **2** | 2,798,410 | 3,330 | 13,956,352 | 0.24 | 3.56 (3.27, 3.88) | 4.52 (4.15, 4.93) | 4.36 (3.995,4.76) |
| **3** | 1,983,643 | 4,072 | 9,850,376 | 0.41 | 5.45 (5.01, 5.93) | 7.52 (6.898, 8.21) | 7.00 (6.417,7.64) |
| **4** | 1,223,426 | 4,975 | 6,045,109 | 0.82 | 9.78 (8.99, 10.64) | 14.36 (13.16, 15.67) | 12.37 (11.33,13.50) |
| **5** | 498,097 | 3,395 | 2,446,328 | 1.39 | 15.81 (14.49, 17.24) | 25.90 (23.64, 28.38) | 20.21 (18.42, 22.16) |
| **At second visit (index year)** |  |  |  |  |  |  |  |
| **0** | 3,198,228 | 518 | 16,048,934 | 0.03 | 1 (ref.) | 1 (ref.) | 1 (ref.) |
| **1** | 3,298,172 | 1,927 | 16,509,307 | 0.12 | 2.44 (2.22, 2.69) | 2.88 (2.61, 3.17) | 2.85 (2.58, 3.14) |
| **2** | 2,776,555 | 3,053 | 13,841,388 | 0.22 | 3.79 (3.45, 4.17) | 5.11 (4.65, 5.62) | 4.80 (4.36, 5.27) |
| **3** | 2,075,598 | 4,077 | 10,304,433 | 0.40 | 6.07 (5.53, 6.65) | 9.06 (8.25, 9.96) | 8.18 (7.44, 8.99) |
| **4** | 1,373,645 | 5,324 | 6,785,802 | 0.78 | 10.82 (9.88, 11.86) | 17.50 (15.93, 19.24) | 14.46(13.15, 15.90) |
| **5** | 588,726 | 3,683 | 2,896,230 | 1.27 | 16.91 (15.40, 18.57) | 32.47 (29.41, 35.85) | 24.81 (22.46, 27.42) |
| Model 1, adjusted for No. of MetS components at 1st visit, age and sex; Model 2, Model 1 plus adjusted for smoking, drinking and exercise; Model 3, Model 2 plus adjusted for estimated GFR. | | | | | | | |
|  | | |  |  |  |  |  |
