# Supplementary material for: Changes in metabolic syndrome status affect the incidence of end-stage renal disease in the general population: a nationwide cohort study
Source: Sci Rep. 2021 Jan 21;11:1957. doi: 10.1038/s41598-021-81396-0 (PMC7820283; doi:10.1038/s41598-021-81396-0)
Supplement: Supplementary file 2 — Supplementary Information. [file 41598_2021_81396_MOESM2_ESM.docx]

**Changes in metabolic syndrome status affect the incidence of end-stage renal disease in the general population: a nationwide cohort study**

Eun Sil Koh^1^, Kyung Do Han^2^, Mee Kyoung Kim^3^, Eun Sook Kim^4^, Min-Kyung Lee^5^, Ga Eun Nam^6^, Oak-Kee Hong^3^ and Hyuk-Sang Kwon^3,*^

^1^Division of Nephrology, Department of Internal Medicine, Yeouido St. Mary’s Hospital, College of Medicine, The Catholic University of Korea, Seoul 07345, Republic of Korea

^2^Department of Statistics and Actuarial Science, Soongsil University, Seoul 06978, Republic of Korea

^3^Division of Endocrinology and Metabolism, Department of Internal Medicine, Yeouido St. Mary’s Hospital, College of Medicine, The Catholic University of Korea, Seoul 07345, Republic of Korea

^4^Division of Endocrinology and Metabolism, Department of Internal Medicine, Incheon St. Mary’s Hospital, College of Medicine, The Catholic University of Korea, Incheon 21431, Republic of Korea

^5^Division of Endocrinology and Metabolism, Department of Internal Medicine, Myongji Hospital, Hanyang University Medical Center, Goyang-Si, Gyeonggi-do 10475, Republic of Korea

^6^Department of Family Medicine, Korea University Anam Hospital, Korea University College of Medicine, Seoul 02841, Republic of Korea

**Word Count:** 3,186 words

**Key words:** Metabolic syndrome; ESRD; Change

***Corresponding author:** Hyuk-Sang Kwon, MD, PhD

Address: Division of Endocrinology and Metabolism, Department of Internal Medicine, Yeouido St. Mary’s Hospital, College of Medicine, The Catholic University of Korea, 10, 63-ro, Yeongdeungpo-gu, Seoul 07345, Republic of Korea

Tel: +82-2-3779-1039, Fax: +82-2-780-3132, E-mail: [drkwon@catholic.ac.kr](mailto:drkwon@catholic.ac.kr)

| **Supplementary Table S2 Adjusted hazard ratios, 95% confidence intervals, and incidence rates of end-stage renal disease development according to the number of MetS components in different renal function groups** | | | | | | | | |
| --- | --- | --- | --- | --- | --- | --- | --- | --- |
| **eGFR (ml/min/1.73 m^2^)** | **No. of MetS compenents** | **N** | **Event (n)** | **Follow-up duration (person-years)** | **Incidence rate (per 1000 person-years)** | Adjusted hazard ratios ( 95% confidence intervals) | | |
|  |  |  |  |  |  | **Model 1** | **Model 2** | **Model 3** |
| **≥ 90** |  |  |  |  |  |  |  |  |
|  | 0 | 1,619,107 | 193 | 8,122,220 | 0.02 | 1 (ref.) | 1 (ref.) | 1 (ref.) |
|  | 1 | 1,517,863 | 320 | 7,596,071 | 0.04 | 1.22 (1.02, 1.46) | 1.33 (1.11,1.60) | 1.33 (1.11, 1.60) |
|  | 2 | 1,166,890 | 395 | 5,817,218 | 0.07 | 1.65 (1.38, 1.96) | 1.93 (1.62, 2.32) | 1.93 (1.62, 2.32) |
|  | 3 | 797,930 | 350 | 3,963,616 | 0.09 | 1.91 (1.60, 2.29) | 2.40 (1.99, 2.90) | 2.40 (1.99, 2.90) |
|  | 4 | 475,892 | 331 | 2,355,574 | 0.14 | 2.75 (2.29, 3.30) | 3.66 (3.01, 4.46) | 3.66 (3.00, 4.45) |
|  | 5 | 181,339 | 161 | 895,143 | 0.18 | 3.40 (2.74,4.21) | 5.02 (3.96, 6.37) | 5.01 (3.95, 6.36) |
| **60-90** |  |  |  |  |  |  |  |  |
|  | 0 | 1,496,983 | 221 | 7,504,175 | 0.03 | 1 (ref.) | 1 (ref.) | 1 (ref.) |
|  | 1 | 1,662,271 | 588 | 8,319,585 | 0.07 | 1.65 (1.42, 1.93) | 1.84 (1.57, 2.15) | 1.83 (1.56, 2.14) |
|  | 2 | 1,472,399 | 870 | 7,343,727 | 0.12 | 2.33 (2.01, 2.70) | 2.84 (2.44, 3.31) | 2.80 (2.40, 3.26) |
|  | 3 | 1,135,247 | 882 | 5,643,754 | 0.16 | 2.80 (2.41, 3.26) | 3.69 (3.16, 4.30) | 3.59 (3.07, 4.18) |
|  | 4 | 761,546 | 1,028 | 3,771,661 | 0.27 | 4.48 (3.86, 5.20) | 6.28 (5.38, 7.34) | 6.05 (5.18, 7.07) |
|  | 5 | 324,571 | 603 | 1,602,985 | 0.38 | 6.08 (5.19, 7.12) | 9.69 (8.17, 11.49) | 9.30 (7.84, 11.03) |
| **< 60** |  |  |  |  |  |  |  |  |
|  | 0 | 82,138 | 104 | 422,540 | 0.25 | 1 (ref.) | 1 (ref.) | 1 (ref.) |
|  | 1 | 118,038 | 1,019 | 593,651 | 1.73 | 6.15 (5.02,7.53) | 7.545 (6.17, 9.24) | 7.94 (6.48,9.72) |
|  | 2 | 137,266 | 1,788 | 680,443 | 2.63 | 9.26 (7.59, 11.29) | 13.33 (10.91, 16.27) | 14.74 (12.07, 17.99) |
|  | 3 | 142,421 | 2,845 | 697,063 | 4.08 | 14.79 (12.14, 18.01) | 23.73 (19.45, 28.96) | 29.05 (23.82, 35.43) |
|  | 4 | 136,207 | 3,965 | 658,567 | 6.02 | 22.60 (18.57, 27.52) | 39.35 (32.25, 48.02) | 50.21 (41.16, 61.26) |
|  | 5 | 82,816 | 2,919 | 398,101 | 7.33 | 30.48 (25.01, 37.16) | 65.96 (53.88, 80.73) | 85.04 (69.47, 104.11) |
| Model 1, adjusted for No. of MetS components at 1st visit, age and sex; Model 2, Model 1 plus adjusted for smoking, drinking and exercise; Model 3, Model 2 plus adjusted for estimated GFR | | | | | | | | |
